# The BiSciCol Triplifier: bringing biodiversity data to the Semantic Web

**DOI:** 10.1186/1471-2105-15-257

**Published:** 2014-07-29

**Authors:** Brian J Stucky, John Deck, Tom Conlin, Lukasz Ziemba, Nico Cellinese, Robert Guralnick

**Affiliations:** Department of Ecology and Evolutionary Biology, University of Colorado, Boulder, Colorado USA; Berkeley Natural History Museums, University of California, Berkeley, California USA; Museum of Natural History, University of Colorado, Boulder, Colorado USA; Florida Museum of Natural History, University of Florida, Gainesville, Florida USA

**Keywords:** Biocollections, Biodiversity informatics, Darwin core, Linked data, Ontology, RDF, Semantic web, SPARQL

## Abstract

**Background:**

Recent years have brought great progress in efforts to digitize the world’s biodiversity data, but integrating data from many different providers, and across research domains, remains challenging. Semantic Web technologies have been widely recognized by biodiversity scientists for their potential to help solve this problem, yet these technologies have so far seen little use for biodiversity data. Such slow uptake has been due, in part, to the relative complexity of Semantic Web technologies along with a lack of domain-specific software tools to help non-experts publish their data to the Semantic Web.

**Results:**

The BiSciCol Triplifier is new software that greatly simplifies the process of converting biodiversity data in standard, tabular formats, such as Darwin Core-Archives, into Semantic Web-ready Resource Description Framework (RDF) representations. The Triplifier uses a vocabulary based on the popular Darwin Core standard, includes both Web-based and command-line interfaces, and is fully open-source software.

**Conclusions:**

Unlike most other RDF conversion tools, the Triplifier does not require detailed familiarity with core Semantic Web technologies, and it is tailored to a widely popular biodiversity data format and vocabulary standard. As a result, the Triplifier can often fully automate the conversion of biodiversity data to RDF, thereby making the Semantic Web much more accessible to biodiversity scientists who might otherwise have relatively little knowledge of Semantic Web technologies. Easy availability of biodiversity data as RDF will allow researchers to combine data from disparate sources and analyze them with powerful linked data querying tools. However, before software like the Triplifier, and Semantic Web technologies in general, can reach their full potential for biodiversity science, the biodiversity informatics community must address several critical challenges, such as the widespread failure to use robust, globally unique identifiers for biodiversity data.

**Electronic supplementary material:**

The online version of this article (doi:10.1186/1471-2105-15-257) contains supplementary material, which is available to authorized users.

## Background

Biocollections represent irreplaceable legacy information about our biosphere that is essential for understanding how biodiversity is changing in an era of unprecedented human impacts [1–3]. Such analyses are only practical if data from biocollections around the world are digitized, integrated, and made widely available online. These tasks are a major focus of the field of biodiversity informatics, and although they present many challenges, they also promise to deliver significant benefits for biodiversity science and its allied disciplines (see, e.g., [4–9]). In recent years, the biodiversity informatics community has made tremendous strides toward achieving this goal by creating shared common vocabularies such as Darwin Core (DwC) [10] and publishing mechanisms such as the Integrated Publishing Toolkit (IPT) [11]. Thanks to these and other national and international initiatives, we now have hundreds of millions of biodiversity records from around the world published in common formats and aggregated into centralized portals for further use.

Along with this success, however, come new challenges for effectively using such a large mass of data. In particular, as the numbers of species, geographic regions, and institutions represented continue to grow, answering questions about the complex interrelationships among these data becomes increasingly difficult. This is due in no small part to the format of most existing data, which have been assembled and mobilized for the Web using relatively “flat,” tabular formats, which often rely on identifiers that are unique only within the context of a given institution or data provider, and which typically have little or no awareness of data from other sources. Aggregation efforts by themselves do not directly solve these problems, so in a very real sense, the biodiversity data landscape still consists of many “islands” of biodiversity data with only limited connectivity. Discovering even simple relationships among these data islands is often prohibitively difficult. For example, a single collecting expedition might ultimately result in a cascade of specimens and their derivatives (tissue samples, genetic data, and so on) scattered across multiple institutional collections. As each institution populates its own data island, the links between these objects are lost, and putting these pieces back together again is, at best, very challenging and at worst, practically impossible.

The Semantic Web (SW) and its core technologies provide a natural solution to these problems by enabling a web of linked data and knowledge where all data objects are uniquely identified and the relationships among them are explicitly defined [12, 13]. Consequently, there is growing recognition of the advantages of linked data technologies not only in biodiversity research and its related disciplines (e.g., [7, 9, 14, 15]) but also throughout the life sciences [16–20].

Despite this considerable interest, most extant biodiversity data remain well outside of the Semantic Web. Why the apparent lack of progress? We identify three major obstacles preventing the more widespread adoption of linked data technologies in biodiversity science. First, the technologies behind the SW are generally much more complex than those of traditional data publishing. Producing high-quality linkable data typically requires, at a minimum, the use of HTTP (HyperText Transfer Protocol) URI (Uniform Resource Identifier)-based identifiers for all data objects, Resource Description Framework (RDF) [21] for representing the data and its interrelationships, and vocabularies and ontologies specified in RDF Schema (RDFS) [22] or the Web Ontology Language (OWL) [23] to describe the kinds of data and relationships that may be used. The traditional tools that these technologies replace, such as relational databases or even spreadsheets, are comparatively much simpler. Second, along with this technological complexity, most available tools for moving data to the SW are either immature, too generic, or require sophisticated knowledge of SW technologies. Such tools are intimidating and unhelpful to users who simply want to publish their data, not become SW experts. Finally, standards for guiding the creation of linked data, such as identifier schemes and services and descriptive ontologies, are either nonexistent, in flux as they undergo active development, or plagued by uncertainty due to competing proposals. This is especially problematic for interdisciplinary fields such as biodiversity science, which spans many knowledge domains, such as taxonomy, genetics, ecology, and geography. However, major collaborative efforts are underway to address this last problem e.g., [24, 25], and we anticipate that robust, stable standards will emerge in the next few years. In the meantime, much more work will be needed to eventually overcome obstacles one and two.

The Biological Sciences Collections (BiSciCol) Triplifier is new software that takes a step toward meeting these challenges by making it easy for biodiversity scientists to take data in traditional tabular representations and transform them into a format suitable for the Semantic Web. The Triplifier accepts data in a variety of common input formats and converts them into a full RDF representation using a consistent RDF vocabulary with RDFS classes and explicit relationships among the class instances. Data in the widely-used Darwin Core-Archive (DwC-A) format [26] are especially easy to process and require the user to have only minimal knowledge of SW technologies. The Triplifier is Free and Open Source software that can be used either as a graphical Web-based application or as a local command-line tool. In this paper, we describe the design and implementation of the Triplifier, summarize its user interface and outputs, discuss the advantages of and potential applications for the Triplifier, and consider the ongoing challenges that currently limit the utility of the Triplifier and other SW technologies in biodiversity science.

## Implementation

In developing the Triplifier, there were four major design goals that guided our work. First, the Triplifier needed to accept biodiversity data in standard tabular formats, including DwC-A, and convert them into a usable, and useful, RDF representation. Second, the software needed to be easy to use, yet flexible enough to handle a variety of input data formats and structures. Third, the Triplifier’s RDF vocabulary should be based primarily on Darwin Core, which has become the standard for representing species occurrence data. Finally, the Triplifier needed to be easily extensible to support new input data sources and formats.

### Software design and architecture

**Figure 1:**
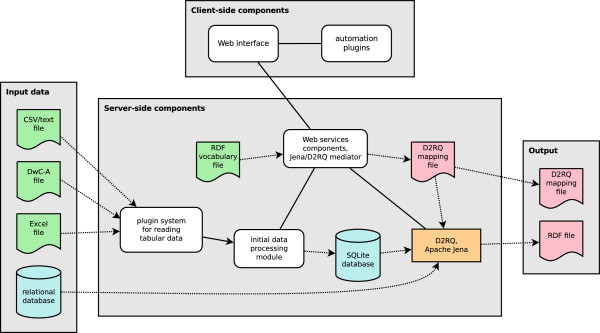
**Simplified architectural block diagram of the Triplifier.** Solid lines indicate connections between major software components, dotted lines indicate movement of data into or out of files and databases, arrowheads indicate the overall direction of data movement through the system. White, rounded boxes represent key Triplifier software components; the orange, rectangular box represents key third-party software components. The remaining symbols represent data files and databases.

To meet these objectives, we chose to build the Triplifier primarily as a dynamic Web application. The software was architected using a typical “Web 2.0” approach, with Ajax (Asynchronous JavaScript and XML)-style lightweight communication between the client and a server backend used for primary data processing. The server-side component of the Triplifier was implemented in Java and communicates with the client by delivering and accepting JSON (JavaScript Object Notation)-formatted data via an HTTP web services API (application programming interface). A simplified diagram of the basic software architecture is presented in Figure 1.

In order to support multiple input formats and to allow the Triplifier to easily support new input formats, the initial stages of data processing in the server software were implemented using a simple plugin architecture. All code that is specific to a particular data format is housed within a single, self-contained Java class that implements a Java interface for reading generic tabular-formatted data. These reader classes are automatically discovered and dynamically loaded by the main server software at run time.

After initial processing, all new incoming data is converted to a standardized representation in a SQLite (http://www.sqlite.org/) database. Most data sources are converted to this database representation with few, or no changes. For Darwin Core Archives, however, we implemented more sophisticated initial processing that takes advantage of this format’s well-defined structure and close relationship with DwC. The column names in a DwC-A are first analyzed to identify which classes are present (see the discussion of the Triplifier data model below) and the data are then “normalized” by moving columns for the different classes into separate tables and eliminating duplicates.

Final output of RDF triples was implemented using D2RQ [27] and Apache Jena [28]. Guided by input from the client interface, the server dynamically builds a D2RQ database-to-RDF mapping that allows the data in the SQLite database to be converted directly to RDF in either N-Triples [29] or Turtle [30] format, or to the DOT format (http://www.graphviz.org/content/dot-language) as a directed graph representation.

The client-side component of the Triplifier was designed to support various degrees of automation depending on the input data format. Conversion of a particular format to RDF may be almost completely automated by writing a JavaScript component that defines how the source data should be mapped to class instances, properties, and relationships in RDF. These JavaScript components must adhere to a simple “interface” and are similar in concept to the plugin system for reading source data on the server. For all input sources, we designed the Web interface to allow the user fine-grained control over the details of how the data are mapped to RDF.

### Vocabulary and data model

As mentioned earlier, a major impediment to the more widespread adoption of SW standards by biodiversity scientists is the lack of robust, standardized RDF vocabularies and ontologies. This was a problem for developing the Triplifier because such standards are needed to produce meaningful and reusable linked data. To solve this problem, we chose to base the Triplifier’s working RDF vocabulary, RDFS class definitions, and ontology primarily on the Darwin Core standard. DwC is by far the most widely used vocabulary for tabular-formatted data, so it made sense to use it as much as possible for the Triplifier.

Even though Darwin Core is available as an RDF document (http://rs.tdwg.org/dwc/rdf/dwcterms.rdf), in its current form it is of limited utility for producing linked data. The most significant shortcomings are confusion about the precise meanings of the DwC “classes” and their associated “ID” terms (e.g., dwc:Occurrence and dwc:OccurrenceID [full expansions for all URI prefixes are given in Table 1]), no statements defining the domains of descriptive properties, and a lack of properties to define the relationships among class instances [10, 31]. We briefly describe how we addressed each of these issues.

**Table 1:** **URI short-form prefixes used in this paper**

Prefix	URI
bsc	http://biscicol.org/terms/biscicol.owl#
dcterms	http://purl.org/dc/terms/
dwc	http://rs.tdwg.org/dwc/terms/
dwcattributes	http://rs.tdwg.org/dwc/terms/attributes/
owl	http://www.w3.org/2002/07/owl#
rdf	http://www.w3.org/1999/02/22-rdf-syntax-ns#
ro	http://www.obofoundry.org/ro/ro.owl#

The principal RDFS classes in the Triplifier’s vocabulary are the DwC “categories,” which are already defined in the DwC RDF as RDFS classes. We interpreted the class-specific “ID” terms as denoting the identifiers that could be used for instances of the DwC classes, which we believe is consistent with current usage of these terms in actual biodiversity datasets. The remaining terms, which are defined in the DwC RDF as type rdf:Property, were included as properties used to connect literal objects (e.g., text strings or numeric values) to class instances. We also included seven RDFS classes defined by the Dublin Core metadata standard [32], one of which is already a part of DwC (dcterms:Location), and the remainder of which are useful for describing biocollections data (dcterms: Agent, dcterms: Image, dcterms: MovingImage, dcterms: PhysicalObject, dcterms: Sound, dcterms: Text).

Although the DwC RDF does not state the domain of any properties, it does include dwcattributes: organizedInClass, which appears to serve a similar function. We interpreted dwcattributes:organizedInClass as defining to which class each property should apply and therefore effectively defining each property’s domain. This worked for nearly all properties except for the so-called “record-level terms,” which do not indicate that they are organized within a single class. Seven of these terms (dcterms:type, dwc: institutionID, dwc: collectionID, dwc: institutionCode, dwc: collectionCode, dwc: ownerInstitutionCode, dwc: basisOfRecord) appeared to most often describe an Occurrence in actual practice, so we considered them as applying to this class. We left the remaining record-level terms in the vocabulary without a single-class domain. Because most of these terms are rarely used, they are not included by default in the Web-based user interface.

As it currently stands, DwC includes virtually no guidance about how class instances should be related to one another in a linked data context, but there have been previous independent efforts to fill this gap, such as the Taxon Concept Ontology [33], the darwin-sw project [34], and the work of the TDWG-RDF interest group (http://tdwg-rdf.googlecode.com/). However, because efforts to develop and standardize full-featured ontologies for biodiversity science and its related disciplines are well under way [25], we chose to develop a limited “ontology” for the Triplifier that defines only four high-level relationships between class instances. This allowed us to move forward with software development while we wait for richer and more descriptive ontologies to become available.

The four properties supported by the Triplifier originated with the broader BiSciCol project and were chosen because they allowed us to capture the essential connections in DwC data. We used the OWL constructs owl: SymmetricProperty and owl: TransitiveProperty to formally define how these properties should be applied when reasoning across multiple RDF statements (see [35] for a detailed discussion of these OWL constructs). The four properties are ro:derives_from (non-symmetric, transitive), bsc:depends_on (non-symmetric, non-transitive), bsc: alias_of (symmetric, transitive), and bsc:related_to (symmetric, non-transitive) (Table 2). These properties had already been selected for the broader BiSciCol project and they allowed us to capture the essential connections in DwC data. The meaning of each property is apparent from its name: ro: derives_from is borrowed from the OBO Relation Ontology [36] and indicates that the subject of an RDF statement was physically derived from the object, bsc: depends_on indicates that the subject could not exist without the object, bsc: alias_of indicates the subject and object are the same thing, and bsc: related_to indicates that the subject and object share a non-dependent relationship.

**Table 2:** **Definitions and examples of the relationship properties used by the Triplifier’s ontology**

Property	Definition	Example	Symmetric	Transitive
obj1 ro: derives_from obj2	Physical material (obj1) that is substantially derived from other physical material (obj2)	A tissue sample (obj1) is derived from a specimen (obj2)	No	Yes
obj1 bsc: depends_on obj2	An entity (obj1) whose existence depends on another entity (obj2)	An identification (obj1) depends on a specimen (obj2)	No	No
obj1 bsc: alias_of obj2	Two instances (obj1, obj2) that are understood to be the same thing	Obj1 and obj2 both refer to the same specimen	Yes	Yes
obj1 bsc: related_to obj2	An entity (obj1) with a non-dependent relationship with another entity (obj2)	A specimen (obj1) is related to a taxon (obj2)	Yes	No

**Figure 2:**
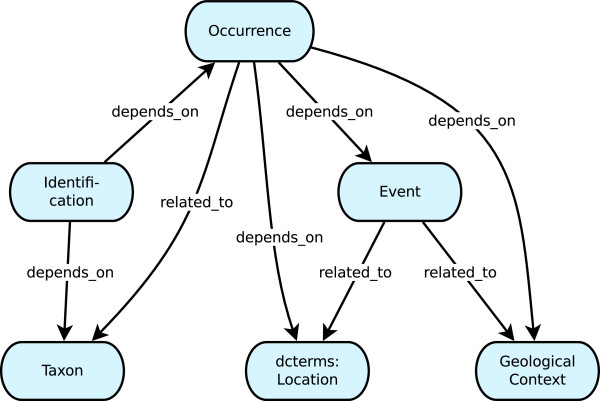
**Diagram of the ontology used by the Triplifier for the six core DwC classes.** For simplicity of presentation, the “dwc” and “bsc” prefixes are omitted.

During the Triplifier’s development, DwC included six core categories or classes (dwc: Occurrence, dwc: Event, dcterms: Location, dwc: GeologicalContext, dwc: Identification, dwc: Taxon), and we focused on explicitly defining the possible relationships among instances of these classes. Recently, a seventh category was added, dwc:MaterialSample [37], but it is not yet included in the Triplifier’s ontology. For each of these six classes, we considered their meanings as defined in the DwC standard as well as how they are most commonly used in practice in order to decide how instances of these classes could best be connected using the four simple properties discussed above. The results of this analysis became the final ontology that we used for developing the Triplifier, which is illustrated in Figure 2. This ontology was intended to cover most, but not all, DwC-based biodiversity data. For example, it does not deal with cases where ro: derives_from might be used, such as a tissue sample that is taken from a whole specimen, and it does not include the use of alias_of. Our experience has been that derives_from is rarely needed when working with current DwC data sets, and alias_of is relevant only for special cases in which a single object has been accidentally assigned multiple identifiers. Thus, the Triplifier does not automatically apply either of these properties by default. However, we designed the Triplifier’s Web interface to allow users to easily express all four relationship properties for their own data, as needed.

### Instance identifiers

A final challenge in developing the Triplifier was the lack of a broadly accepted and widely used standard for generating and resolving identifiers for biodiversity data. Although several options are available, such as Archival Resource Keys (ARKs), Biocode Commons Identifiers (BCIDs), Digital Object Identifiers (DOIs), Life Sciences Identifiers (LSIDs), Uniform Resource Name (URN)-based mechanisms, and others [38–40], none have been widely adopted by biodiversity data providers [7, 41]. Given this reality, we decided to neither enforce nor endorse any particular identifier standard in the Triplifier and to instead allow users to work directly with whatever identifiers they prefer.

Many data, however, have only locally unique identifiers, which are not useful for linking on the SW, and some identifiers are not even unique within a single dataset (e.g., integer keys that are reused across database tables). Moreover, flat, single-table input formats such as DwC-A usually have no identifiers at all for most of the class instances that are implicitly present in the data. To handle these cases, we implemented a simple identifier construction algorithm. If the user does not indicate that input data uses globally unique identifiers, the Triplifier generates identifiers for each class instance by concatenating three pieces of information: SQLite_database_table_name + “.” + identifier_column_name + “_” + local_identifier. In the case of DwC-As, if identifiers for a particular class are missing entirely, local integer-based identifiers are generated during the data normalization step. It should be noted that this scheme is only guaranteed to produce identifiers that are unique within a given version of a dataset, which is sufficient for using the final RDF by itself but not for linking it with other datasets. Although we considered having the Triplifier mint new globally unique identifiers for user data, we ultimately decided that this was beyond the software’s intended scope.

### Active development

We are currently developing a command-line version of the Triplifier to complement the Web-based Triplifier. The command-line Triplifier is intended for efficient, high-throughput processing of large numbers of data files or very large data files. Its user interface features two basic modes of operation. First, users can supply a custom D2RQ mapping file to guide the conversion of the source data to RDF. Mapping files can initially be generated by the Web-based Triplifier, further modified as needed, then used with the command-line Triplifier for batch data processing. The second mode of operation uses custom Java classes that fully automate the data conversion process for specific input data formats. These classes are conceptually analogous to the automation plugins described above for the Web-based Triplifier, and when the command-line tool is used in this mode, no D2RQ mapping file is required.

Automation classes for the command-line Triplifier can also take advantage of a Java class framework that was designed to make it easy to customize how source data are converted to RDF. For instance, automation classes could use terms from alternative vocabularies or ontologies, allowing for a broader range of semantic interpretations of source data files, or they could incorporate custom globally unique identifier schemes. Support for these relatively low-level modifications goes beyond what is easily achievable through the Web-based interface.

## Results

The BiSciCol Triplifier is available to users as both a Web application [42] and a command-line program. The Triplifier is Free and Open Source Software and all source code is provided under the terms of the BSD 3-Clause License [43] at the Triplifier’s project site [44]. Pre-built executables of the command-line tool are available via the Triplifier’s Subversion repository. The project site also includes user and developer documentation, and additional information about the philosophy and design decisions that guided Triplifier development can be found on the BiSciCol blog [45].

The Triplifier currently accepts source data in a variety of common tabular data formats, including comma-separated values (CSV) text files, OpenDocument (OpenOffice.org and LibreOffice) and Microsoft Excel spreadsheets, and DwC-A. The Triplifier also supports direct network connections to popular relational database management systems such as PostgreSQL, MySQL, Oracle Database, and Microsoft SQL Server. Input data are not required to follow any particular structure and are not required to use DwC terms.

After loading an input data source, the user must provide the information about the data that the Triplifier needs to successfully convert them to RDF. With the Web interface, this requires four steps. First, if the source includes multiple data tables, the user indicates which keys join the tables together (in the sense of relational databases). Second, the user specifies which table columns should be used as identifiers for instances of the classes in the Triplifier’s vocabulary and whether those columns contain globally unique identifiers. Third, columns with literal data are matched to property names in the Triplifier’s vocabulary and the classes they describe. Fourth, the user indicates how class instances should be connected to one another with the four Triplifier relationship properties.

Upon completion of these four steps, the Triplifier can generate and return the RDF representation of the user’s data as a single N-Triples or Turtle-formatted text file. Alternatively, the triples can be converted to the DOT graph description format and downloaded as a DOT file, which allows the data to be visualized with software such as Graphviz [46]. The user may also download the dynamically generated mapping file that captures all of the information provided in the four configuration steps. These mapping files can then be used with the command-line version of the Triplifier to rapidly process larger volumes of data.

For source data that is in the format of a DwC-A, the process is even simpler. If the user chooses, the Triplifier can automatically analyze the source archive and complete all four of the configuration steps with no user intervention. At that point, the user can either customize the configuration as desired or simply request the RDF file. In most cases, running DwC-As through the Triplifier is as straightforward as uploading the archive, then downloading the RDF representation. Example RDF output from the Triplifier for typical DwC-A data, including a graphical representation of the generated RDF triples, is provided in Additional file 1.

We built the Triplifier with biodiversity data in mind, but the code follows a modular design and should be, for the most part, relatively easy to adapt to other knowledge domains. For example, all that is required to support a new input data format is writing a single Java class that implements a reader plugin for the Triplifier’s server-side data processing system. No existing code needs to be modified. Customizing or replacing the Triplifier’s RDF vocabulary is also straightforward because it is defined by a single RDFS file.

## Discussion

The BiSciCol Triplifier is not the first software for converting tabular data to RDF see, e.g., [47–49], but for biodiversity scientists, there are at least three key advantages that separate the Triplifier from most of these other tools. First, unlike other software which often works with only one or a few input formats, the Triplifier supports a broad range of data formats, ranging from plain text files to full-fledged databases, and it is the only tool of which we are aware that directly supports DwC-As. Second, the Triplifier is specifically tailored for biodiversity data and comes with a ready-to-use vocabulary and interface based upon DwC. Third, and perhaps most important, because of its domain-specificity, the Triplifier can hide most or all of the complexity of SW technologies from the end user. For most users, and for most kinds of data, using the Triplifier will require little more than a conceptual understanding of the principles behind linked data technologies. For users with data in DwC-As, conversion to RDF can be fully automated and requires no special knowledge at all. This is in contrast to more generic tabular data converters for which the user must specify complex mappings that require detailed knowledge of the target RDF vocabularies and ontologies.

Despite these advantages, the current landscape for linked data in biodiversity science will likely limit the Triplifier’s ability to bring biodiversity data to the SW, at least in the short term. There are two main reasons for this. First, the absence of robust, standardized, and widely-accepted vocabularies and ontologies for linkable biodiversity data means that the RDF generated by the Triplifier is neither as expressive nor as broadly useful as it could be. For example, we do not envision the Triplifier’s simplistic ontology for relationships among its class instances as a long-term solution. Rather, we consider it merely a means for moving forward until a more satisfactory ontology becomes available and accepted as a standard.

Second, the anarchy presently governing the use of identifiers in biodiversity data is a major impediment to using these data, and RDF generated for them by the Triplifier, on the SW. If the dream of making all biodiversity data universally accessible and linkable is to become reality, biodiversity data must use identifiers that are globally unique, resolvable, and above all, persistent [7]. The Triplifier takes steps to ensure that identifiers in the data it processes are unique within the dataset, which is sufficient for producing functional RDF, but data without permanent and globally unique identifiers cannot be usefully linked with data from other sources. Unfortunately, a permanent solution to this challenge does not seem near at hand, and finding such a solution should be an urgent community priority.

Nevertheless, there are several ways in which the Triplifier will still be useful to biodiversity scientists with data in traditional formats, even if those data are not immediately destined for the larger SW. Perhaps most important, RDF-formatted data from the Triplifier can be aggregated in either local or remote “triple stores” (databases specialized for storing RDF statements) and examined with SW querying tools such as SPARQL [50]. Analyzing DwC-based biodiversity data in this way, rather than as rows in a spreadsheet or relational database table, often makes it much easier to answer high-level questions about the data. For example, suppose we would like to know which Occurrence instances in a DwC dataset are associated with taxonomic information. In a tabular context, answering this conceptually simple question is quite cumbersome and could require inspecting the values of more than 20 table columns. With the RDF output generated by the Triplifier, we need only ask which Occurrence instances are related to a Taxon instance, and this question can be concisely represented by a simple SPARQL query, such as:

SELECT ?occurrence

WHERE {

?occurrence bsc:related_to ?taxon.

?occurrence a dwc:Occurrence.

?taxon a dwc:Taxon

}.

Thus, unlike traditional relational databases, RDF makes it possible to work directly at the level of class instances and their relationships. This can greatly simplify the process of translating questions about the data into the queries that will answer them, and the benefits of this approach have already been demonstrated in other life sciences domains e.g., [51].

We also envision the Triplifier as a hands-on tool for biodiversity researchers to learn about and experiment with SW technologies. In this role, it should be especially useful to those who are are curious about moving their data to the SW but have so far been deterred by the complexity of the SW technology stack. With the Triplifier, such researchers can easily apply core SW technologies directly to their own data and study the results. Use of the familiar DwC terms in the Triplifier’s RDF vocabulary and support for visualization software such as Graphviz will further assist researchers in interpreting and understanding the RDF representations of their data.

## Conclusions

The BiSciCol Triplifier is new open-source software that automates the process of converting tabular-format biodiversity data to RDF suitable for use on the SW. The Triplifier offers the flexibility of both Web-based and command-line interfaces, supports a wide variety of common input formats, including the popular DwC-A format, and comes with a vocabulary and simple ontology based on the widely-used Darwin Core standard. Output formats include Turtle and N-Triples for RDF and DOT for graph visualizations. A modular design makes the Triplifier adaptable to other input data formats, RDF vocabularies, and research domains.

The BiSciCol Triplifier makes it easier than ever before for biodiversity scientists to apply modern SW technologies to their data. Expressing tabular-format biodiversity data as RDF is useful not only because it allows the data to link with other data sources on the SW, but also because it allows researchers to use powerful query tools such as SPARQL to answer complex questions about their data. The Triplifier does not require users to have extensive knowledge of RDF, RDFS vocabularies, or OWL ontologies, which should make it a valuable aid for biodiversity researchers who wish to learn about and experiment with these technologies.

The Semantic Web holds great potential for biodiversity science, but a variety of challenges continue to make actually achieving this potential elusive. Perhaps most critically, biodiversity data continue to suffer from the widespread use of identifiers that are neither persistent nor globally unique, and this severely limits the usefulness of these data on the global SW. Better methods for visualizing linked biodiversity data are also a pressing need, especially for biodiversity scientists who are just beginning to explore how their data might work in a linked context. Looking further ahead, as the Triplifier and other, complementary efforts eventually succeed in mobilizing biodiversity data for the SW, we will likely need new computational tools to make sense of such a massive, and massively interconnected, dataset.

First, though, we need to make it less difficult for biodiversity researchers to actually get their data to the SW in the first place, and the BiSciCol Triplifier is a significant step toward this goal. Our hope is that definitive new standards for vocabularies, ontologies, and identifiers, in concert with software tools like the Triplifier, will make SW technologies as easy to use in the future as databases and spreadsheets have been in the past.

## Availability and requirements

**Project name:** BiSciCol Triplifier

**Project home page:**http://www.biscicol.org/triplifier/ (Web-based application); http://triplifier.googlecode.com/ (project site, documentation, and source code)

**Operating system(s):** The Web-based interface only requires a modern web browser and has been tested with recent versions of Firefox, Chrome, Opera, Safari, and Internet Explorer. The command-line Triplifier and server-side software require Java and should run on all modern operating systems for which the Java run-time environment and development tools are available (e.g., GNU/Linux, Windows, OSX).

**Programming language:** Java and JavaScript

**Other requirements:** The server-side software requires a Java web application server such as Apache Tomcat or GlassFish. All other required components are included with the source distribution.

**License:** BSD 3-Clause License (http://opensource.org/licenses/BSD-3-Clause)

**Any restrictions to use by non-academics:** Non-academics may freely use this software.

## Electronic supplementary material

Additional file 1: **Supplementary material for “The BiSciCol Triplifier: bringing biodiversity data to the Semantic Web”.** The supplementary material provides RDF output generated by the Triplifier for a small input DwC-A data set, and includes a graphical representation of the RDF output. (PDF 630 KB)

## References

[1] Moritz C, Patton JL, Conroy CJ, Parra JL, White GC, Beissinger SR (2008). Impact of a century of climate change on small-mammal communities in Yosemite National Park, USA. Science.

[2] Scoble M (2010). Rationale and value of natural history collections digitisation. Biodivers Inform.

[3] Erb LP, Ray C, Guralnick R (2011). On the generality of a climate-mediated shift in the distribution of the American pika (*Ochotona princeps*). Ecology.

[4] Bisby FA (2000). The quiet revolution: biodiversity informatics and the Internet. Science.

[5] Godfray HCJ, Clark BR, Kitching IJ, Mayo SJ, Scoble MJ (2007). The Web and the structure of taxonomy. Syst Biol.

[6] Sarkar IN (2007). Biodiversity informatics: organizing and linking information across the spectrum of life. Brief Bioinform.

[7] Page RDM (2008). Biodiversity informatics: the challenge of linking data and the role of shared identifiers. Brief Bioinform.

[8] Guralnick R, Hill A (2009). Biodiversity informatics: automated approaches for documenting global biodiversity patterns and processes. Bioinformatics.

[9] Parr CS, Guralnick R, Cellinese N, Page RDM (2012). Evolutionary informatics: unifying knowledge about the diversity of life. Trends Ecol Evol.

[10] Wieczorek J, Bloom D, Guralnick R, Blum S, Döring M, Giovanni R, Robertson T, Vieglais D (2012). Darwin core: an evolving community-developed biodiversity data standard. PLoS One.

[11] Robertson T, Döring M, Guranick R, Bloom D, Braak K, Otegui J, Russell L, Wieczorek J, Desmet P (2014). The GBIF integrated publishing toolkit: facilitating the efficient publishing of biodiversity data on the internet. PLoS One.

[12] Berners-Lee T, Hendler J, Lassila O (2001). The semantic web. Sci Am.

[13] Heath T, Bizer C (2011). Linked data: evolving the web into a global data space. Synth Lect Semantic Web Theory Technol.

[14] Madin JS, Bowers S, Schildhauer MP, Jones MB (2008). Advancing ecological research with ontologies. Trends Ecol Evol.

[15] Deans AR, Yoder MJ, Balhoff JP (2012). Time to change how we describe biodiversity. Trends Ecol Evol.

[16] Stevens R (2000). Ontology-based knowledge representation for bioinformatics. Brief Bioinform.

[17] Blake JA, Bult CJ (2006). Beyond the data deluge: data integration and bio-ontologies. J Biomed Inform.

[18] Good BM, Wilkinson MD (2006). The life sciences semantic web is full of creeps!. Brief Bioinform.

[19] Antezana E, Kuiper M, Mironov V (2009). Biological knowledge management: the emerging role of the Semantic Web technologies. Brief Bioinform.

[20] Chen H, Yu T, Chen JY (2013). Semantic web meets integrative biology: a survey. Brief Bioinform.

[21] **RDF primer** http://www.w3.org/TR/2004/REC-rdf-primer-20040210/

[22] **RDF Vocabulary Description Language 1.0: RDF Schema** http://www.w3.org/TR/rdf-schema/

[23] **OWL 2 Web Ontology Language primer (second edition)** http://www.w3.org/TR/2012/REC-owl2-primer-20121211/

[24] Wooley JC, Field D, Glöckner F-O (2009). Extending standards for genomics and metagenomics data: a research coordination network for the genomic standards consortium (RCN4GSC). Stand Genomic Sci.

[25] Walls RL, Deck J, Guralnick R, Baskauf S, Beaman R, Blum S, Bowers S, Buttigieg PL, Davies N, Endresen D, Gandolfo MA, Hanner R, Janning A, Krishtalka L, Matsunaga A, Midford P, Morrison N, Tuama ÉÓ, Schildhauer M, Smith B, Stucky BJ, Thomer A, Wieczorek J, Whitacre J, Wooley J (2014). Semantics in support of biodiversity knowledge discovery: an introduction to the biological collections ontology and related ontologies. PLoS One.

[26] Robertson T, Döring M, Wieczorek J, De Giovanni R, Vieglais D: **Darwin Core text guide.** http://rs.tdwg.org/dwc/terms/guides/text/10.1371/journal.pone.0029715 PMC3253084 22238640

[27] Bizer C, Seaborne A: **D2RQ – treating non-RDF databases as virtual RDF graphs.** http://iswc2004.semanticweb.org/posters/PID-SMCVRKBT-1089637165.pdf

[28] McBride B (2002). Jena: a Semantic Web toolkit. IEEE Internet Comput.

[29] Beckett D: **RDF 1.1 N-Triples: A line-based syntax for an RDF graph.** http://www.w3.org/TR/n-triples/

[30] Beckett D, Berners-Lee T: **Turtle - Terse RDF Triple Language.** http://www.w3.org/TeamSubmission/turtle/

[31] Baskauf SJ, Webb CO: **Rationale for a Semantic Web implementation of Darwin Core.** http://code.google.com/p/darwin-sw/wiki/Rationale

[32] Weibel S (1997). The Dublin core: a simple content description model for electronic resources. Bull Am Soc Inf Sci Technol.

[33] **TaxonConcept: species concepts for the Semantic Web** http://www.taxonconcept.org/ontologies/

[34] Webb C, Baskauf S: D-SW: **Darwin Core data for the Semantic Web.** http://www.tdwg.org/fileadmin/2011conference/slides/Webb_DarwinSW.pdf

[35] Allemang D, Hendler JA (2008). Semantic Web for the Working Ontologist: Modeling in RDF, RDFS and OWL.

[36] Smith B, Ceusters W, Klagges B, Köhler J, Kumar A, Lomax J, Mungall C, Neuhaus F, Rector AL, Rosse C (2005). Relations in biomedical ontologies. Genome Biol.

[37] **DarwinCore Issue 167: MaterialSample** https://code.google.com/p/darwincore/issues/detail?id=167

[38] Martin S, Hohman MM, Liefeld T (2005). The impact of Life Science Identifier on informatics data. Drug Discov Today.

[39] Hilse H-W, Kothe J, Consortium of European Research Libraries, European Commission on Preservation and Access (2006). Implementing Persistent Identifiers: Overview of Concepts, Guidelines and Recommendations.

[40] **Biocode Commons Identifiers (BCIDs)** http://bcid.googlecode.com/

[41] Chavan VS, Ingwersen P (2009). Towards a data publishing framework for primary biodiversity data: challenges and potentials for the biodiversity informatics community. BMC Bioinformatics.

[42] **Triplifier Web Application** http://www.biscicol.org/triplifier/

[43] **The BSD 3-Clause License** http://opensource.org/licenses/BSD-3-Clause

[44] **BiSciCol Triplifier Project Site** http://triplifier.googlecode.com/

[45] **The BiSciCol Blog** http://biscicol.blogspot.com/

[46] Ellson J, Gansner ER, Koutsofios E, North SC, Woodhull G, Jünger M, Mutzel P (2004). Graphviz and Dynagraph — static and dynamic graph drawing tools. Graph Draw Softw.

[47] Han L, Finin T, Parr C, Sachs J, Joshi A, Sheth A, Staab S, Dean M, Paolucci M, Maynard D, Finin T, Thirunarayan K (2008). RDF123: From Spreadsheets to RDF. Semantic Web - ISWC 2008. Volume 5318.

[48] Langegger A, Wöß W, Bernstein A, Karger DR, Heath T, Feigenbaum L, Maynard D, Motta E, Thirunarayan K (2009). XLWrap – Querying and Integrating Arbitrary Spreadsheets with SPARQL. Semantic Web - ISWC 2009. Volume 5823.

[49] Lebo T, Erickson JS, Ding L, Graves A, Williams GT, DiFranzo D, Li X, Michaelis J, Zheng JG, Flores J, Shangguan Z, McGuinness DL, Hendler J, Wood D (2011). Producing and Using Linked Open Government Data in the TWC LOGD Portal. Link Gov Data.

[50] **SPARQL Query Language for RDF** http://www.w3.org/TR/rdf-sparql-query/

[51] Asiaee AH, Doshi P, Minning T, Sahoo S, Parikh P, Sheth A, Tarleton RL, Baker CJO, Butler G, Jurisica I (2013). From questions to effective answers: on the utility of knowledge-driven querying systems for life sciences data. Data Integr Life Sci. Volume 7970.

