# Supplementary material for: The BiSciCol Triplifier: bringing biodiversity data to the Semantic Web
Source: BMC Bioinformatics. 2014 Jul 29;15(1):257. doi: 10.1186/1471-2105-15-257 (PMC4124153; doi:10.1186/1471-2105-15-257)
Supplement: Supplementary file 1 — Additional file 1: Supplementary material for “The BiSciCol Triplifier: bringing biodiversity data to the Semantic Web”. The supplementary material provides RDF output generated by the Triplifier for a small input DwC-A data set, and includes a graphical representation of the RDF output. (PDF 630 KB) [file 12859_2014_6524_MOESM1_ESM.pdf]

Supplementary material for
"The BiSciCol Triplifier: bringing biodiversity data to the Semantic Web"

To illustrate how the Triplifier converts DwC data to RDF, this supplementary material includes a small input DwC-A data set (Table S1), the Turtle-formatted RDF output that was automatically generated by the Triplifier from the input DwC-A data (Listing S1), and a graphical representation of the RDF output (Figure S1). The input data were taken from the digitized field notes of Junius Henderson, which are available at http://ipt.vertnet.org:8080/ipt/resource.do?r=hendersonnotebooks1-3.

**id:**	
**occurrenceStatus:**	present
**locality:**	Boulder, Colorado
**collectionCode:**	HendersonNotes
**month:**	7
**verbatimEventDate:**	28-Jul-05
**phylum:**	Chordata
**associatedMedia:**	http://en.wikisource.org/wiki/Page:Field_ …
**modified:**	3/15/2012
**genus:**	*Sayornis*
**basisOfRecord:**	HumanObservation
**catalogNumber:**	JHFN1-3-A
**eventDate:**	7/28/1905
**identifiedBy:**	Gaurav
**identificationRemarks:**	Observation cross-referenced using ITIS …
**taxonRemarks:**	ITIS taxonomic serial number (TSN) used …
**taxonomicStatus:**	valid
**day:**	28
**countryCode:**	US
**country:**	United States of America
**fieldNotes:**	http://en.wikisource.org/wiki/Field_Notes_of …
**recordedBy:**	Junius Henderson
**family:**	Tyrannidae
**year:**	1905
**institutionCode:**	UCM
**vernacularName:**	Say's Phoebe
**verbatimLocality:**	Boulder, Colo
**scientificName:**	*Sayornis saya* (Bonaparte, 1825)
**order:**	Passeriformes
**kingdom:**	Animalia
**class:**	Aves
**dateIdentified:**	7/28/1905

Table S1. A single DwC-A data record used as demonstration input for the Triplifier. These data are the first record from the DwC-A of the complete Henderson field notes. Some long data values were truncated to save space, with missing text indicated by an ellipsis ("…").

```
@prefix rdfs:    <http://www.w3.org/2000/01/rdf-schema#> .
@prefix ro:      <http://www.obofoundry.org/ro/ro.owl#> .
@prefix owl:     <http://www.w3.org/2002/07/owl#> .
@prefix xsd:     <http://www.w3.org/2001/XMLSchema#> .
@prefix rdf:     <http://www.w3.org/1999/02/22-rdf-syntax-ns#> .
@prefix bsc:     <http://biscicol.org/terms/biscicol.owl#> .

<http://rs.tdwg.org/dwc/terms/countryCode>
      a       rdf:Property .

<http://rs.tdwg.org/dwc/terms/collectionCode>
      a       rdf:Property .

<http://rs.tdwg.org/dwc/terms/year>
      a       rdf:Property .

<http://rs.tdwg.org/dwc/terms/Identification>
      a       rdfs:Class .

<http://rs.tdwg.org/dwc/terms/catalogNumber>
      a       rdf:Property .

<http://rs.tdwg.org/dwc/terms/recordedBy>
      a       rdf:Property .

<urn:x-biscicol:maintable.identificationID_1>
      a       <http://rs.tdwg.org/dwc/terms/Identification> ;
      bsc:depends_on <urn:x-biscicol:maintable.occurrenceID_1> , <urn:x-
biscicol:taxon.id_1> ;
      <http://rs.tdwg.org/dwc/terms/dateIdentified>
              "1905-07-28" ;
      <http://rs.tdwg.org/dwc/terms/identificationRemarks>
              "Observation cross-referenced using ITIS ..." ;
      <http://rs.tdwg.org/dwc/terms/identifiedBy>
              "Gaurav" .

<http://rs.tdwg.org/dwc/terms/institutionCode>
      a       rdf:Property .

<http://rs.tdwg.org/dwc/terms/Occurrence>
      a       rdfs:Class .

<http://rs.tdwg.org/dwc/terms/Event>
      a       rdfs:Class .

<http://rs.tdwg.org/dwc/terms/identificationRemarks>
      a       rdf:Property .

<http://rs.tdwg.org/dwc/terms/vernacularName>
      a       rdf:Property .

<http://rs.tdwg.org/dwc/terms/kingdom>
      a       rdf:Property .

<urn:x-biscicol:maintable.occurrenceID_1>
      a       <http://rs.tdwg.org/dwc/terms/Occurrence> ;
```

```
      bsc:depends_on <urn:x-biscicol:location.id_1> , <urn:x-
biscicol:event.id_1> ;
      bsc:related_to <urn:x-biscicol:taxon.id_1> ;
      <http://rs.tdwg.org/dwc/terms/associatedMedia>
              "http://en.wikisource.org/wiki/Page:Field_Notes_..." ;
      <http://rs.tdwg.org/dwc/terms/basisOfRecord>
              "HumanObservation" ;
      <http://rs.tdwg.org/dwc/terms/catalogNumber>
              "JHFN1-3-A" ;
      <http://rs.tdwg.org/dwc/terms/collectionCode>
              "HendersonNotes" ;
      <http://rs.tdwg.org/dwc/terms/institutionCode>
              "UCM" ;
      <http://rs.tdwg.org/dwc/terms/occurrenceStatus>
              "present" ;
      <http://rs.tdwg.org/dwc/terms/recordedBy>
              "Junius Henderson" .

<http://rs.tdwg.org/dwc/terms/Taxon>
      a       rdfs:Class .

<http://rs.tdwg.org/dwc/terms/taxonRemarks>
      a       rdf:Property .

<http://rs.tdwg.org/dwc/terms/occurrenceStatus>
      a       rdf:Property .

<http://rs.tdwg.org/dwc/terms/family>
      a       rdf:Property .

<http://rs.tdwg.org/dwc/terms/country>
      a       rdf:Property .

<urn:x-biscicol:event.id_1>
      a       <http://rs.tdwg.org/dwc/terms/Event> ;
      bsc:related_to <urn:x-biscicol:location.id_1> ;
      <http://rs.tdwg.org/dwc/terms/day>
              "28" ;
      <http://rs.tdwg.org/dwc/terms/eventDate>
              "1905-07-28" ;
      <http://rs.tdwg.org/dwc/terms/fieldNotes>
              "http://en.wikisource.org/wiki/Field_Notes_of_Junius_Henderson" ;
      <http://rs.tdwg.org/dwc/terms/month>
              "7" ;
      <http://rs.tdwg.org/dwc/terms/verbatimEventDate>
              "July 28, 1905" ;
      <http://rs.tdwg.org/dwc/terms/year>
              "1905" .

<http://rs.tdwg.org/dwc/terms/basisOfRecord>
      a       rdf:Property .

<http://rs.tdwg.org/dwc/terms/identifiedBy>
      a       rdf:Property .

<http://rs.tdwg.org/dwc/terms/fieldNotes>
      a       rdf:Property .
```

```
<http://purl.org/dc/terms/Location>
      a       rdfs:Class .

<http://rs.tdwg.org/dwc/terms/verbatimEventDate>
      a       rdf:Property .

<http://rs.tdwg.org/dwc/terms/DataSet>
      a       rdfs:Class .

bsc:related_to
      a       rdf:Property .

<http://rs.tdwg.org/dwc/terms/dateIdentified>
      a       rdf:Property .

<http://rs.tdwg.org/dwc/terms/scientificName>
      a       rdf:Property .

<http://rs.tdwg.org/dwc/terms/month>
      a       rdf:Property .

<http://rs.tdwg.org/dwc/terms/order>
      a       rdf:Property .

<http://rs.tdwg.org/dwc/terms/class>
      a       rdf:Property .

<http://rs.tdwg.org/dwc/terms/genus>
      a       rdf:Property .

<http://rs.tdwg.org/dwc/terms/eventDate>
      a       rdf:Property .

<http://rs.tdwg.org/dwc/terms/taxonomicStatus>
      a       rdf:Property .

<urn:x-biscicol:henderson-short.zip>
      a       <http://rs.tdwg.org/dwc/terms/DataSet> ;
      bsc:related_to <urn:x-biscicol:maintable.occurrenceID_1> .

<http://rs.tdwg.org/dwc/terms/verbatimLocality>
      a       rdf:Property .

<http://rs.tdwg.org/dwc/terms/locality>
      a       rdf:Property .

<urn:x-biscicol:taxon.id_1>
      a       <http://rs.tdwg.org/dwc/terms/Taxon> ;
      <http://rs.tdwg.org/dwc/terms/class>
              "Aves" ;
      <http://rs.tdwg.org/dwc/terms/family>
              "Tyrannidae" ;
      <http://rs.tdwg.org/dwc/terms/genus>
              "Sayornis" ;
      <http://rs.tdwg.org/dwc/terms/kingdom>
              "Animalia" ;
```

```
      <http://rs.tdwg.org/dwc/terms/order>
              "Passeriformes" ;
      <http://rs.tdwg.org/dwc/terms/phylum>
              "Chordata" ;
      <http://rs.tdwg.org/dwc/terms/scientificName>
              "Sayornis saya (Bonaparte, 1825)" ;
      <http://rs.tdwg.org/dwc/terms/taxonRemarks>
              "ITIS taxonomic serial number (TSN) used ..." ;
      <http://rs.tdwg.org/dwc/terms/taxonomicStatus>
              "valid" ;
      <http://rs.tdwg.org/dwc/terms/vernacularName>
              "Say's Phoebe" .

bsc:depends_on
      a       rdf:Property .

<http://rs.tdwg.org/dwc/terms/phylum>
      a       rdf:Property .

<http://rs.tdwg.org/dwc/terms/associatedMedia>
      a       rdf:Property .

<urn:x-biscicol:location.id_1>
      a       <http://purl.org/dc/terms/Location> ;
      <http://rs.tdwg.org/dwc/terms/country>
              "United States of America" ;
      <http://rs.tdwg.org/dwc/terms/countryCode>
              "US" ;
      <http://rs.tdwg.org/dwc/terms/locality>
              "Boulder, Colorado" ;
      <http://rs.tdwg.org/dwc/terms/verbatimLocality>
              "Boulder, Colo" .

<http://rs.tdwg.org/dwc/terms/day>
      a       rdf:Property .
```

Listing S1. Turtle-formatted RDF output automatically generated by the Triplifier from the DwC data in Table S1. Some long data values have been truncated to improve readability, with missing text indicated by an ellipsis ("…").

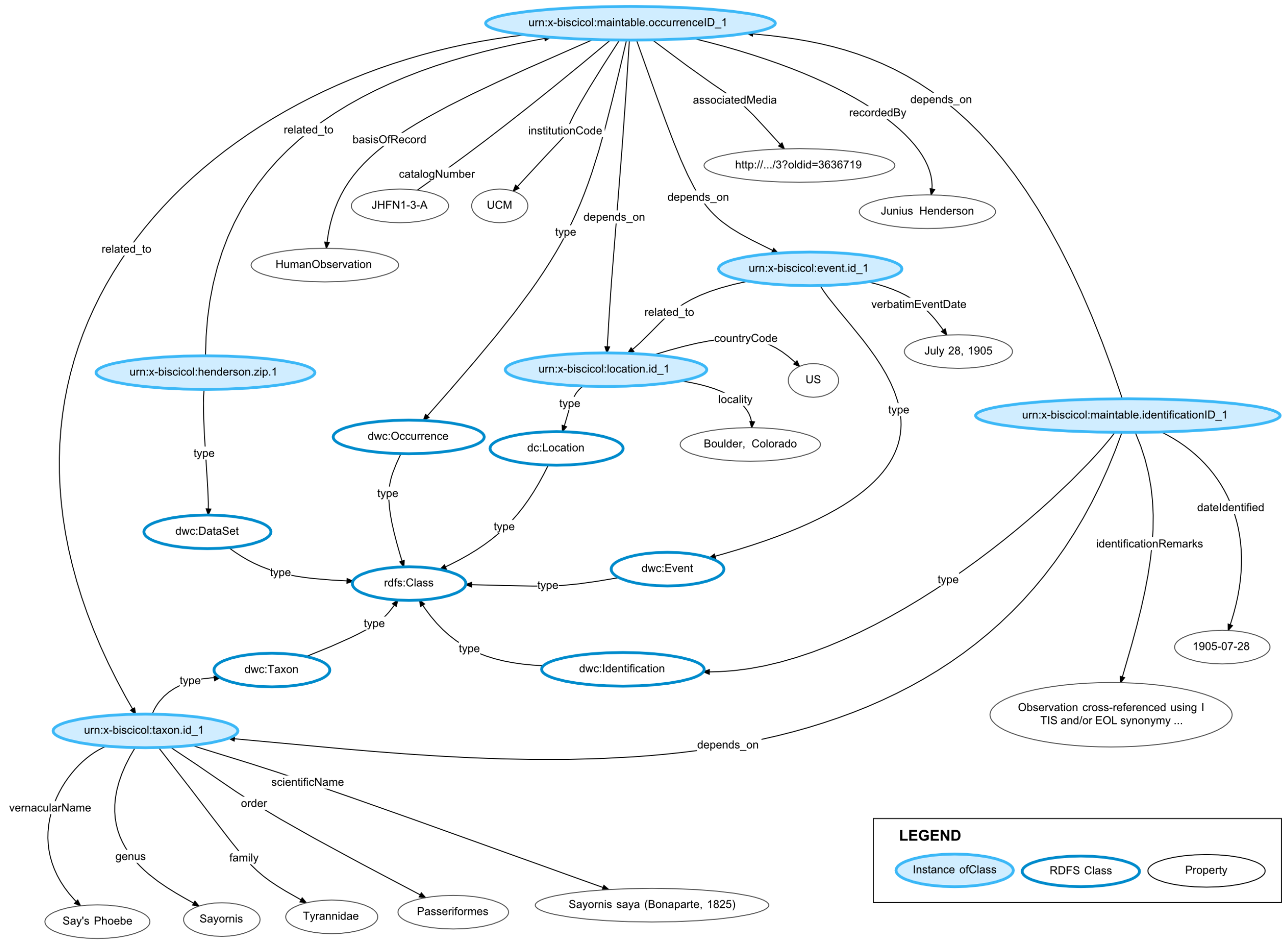

Figure S1. Graphical representation of the RDF statements generated by the Triplifier for the DwC data in Table S1. White ellipses with blue outlines represent RDFS classes, light blue ellipses with blue outlines represent unique instances of RDFS classes, and white ellipses with black outlines represent properties associated with RDFS class instances. To avoid diagram clutter, not all of the properties output in Listing S1 are included in this graphical representation.
